# Multi-Objective NSGA-II Optimization for Broadband Beamforming with Spherical Harmonic Domain Assistance

**DOI:** 10.3390/s23208403

**Published:** 2023-10-12

**Authors:** Zhenghong Liu, Haocheng Zhou, Xiyu Song, Mei Wang, Liuqing Weng

**Affiliations:** 1 School of Information and Communication, Guilin University of Electronic Technology, Guilin 541004, Chinaz2909719840@163.com (H.Z.);; 2 Ministry of Education Key Laboratory of Cognitive Radio and Information Processing, Guilin 541004, China; 3 School of Information Science & Engineering, Guilin University of Technology, Guilin 541006, China

**Keywords:** array processing, multi-objective optimization, sidelobe suppression, pareto-optimal, white noise gain

## Abstract

Sidelobe suppression is a major challenge in wideband beamforming for acoustic research, especially in high noise and reverberation environments. In this paper, we propose a multi-objective NSGA-II wideband beamforming method based on a spherical harmonic domain for spherical microphone arrays topology. The method takes white noise gain, directional index and maximum sidelobe level as the optimization objectives of broadband beamforming, adopts the NSGA-II optimization strategy with constraints to estimate the Pareto optimal solution, and provides three-dimensional broadband beamforming capability. Our method provides superior sidelobe suppression across different spherical harmonic orders compared to commonly used multi-constrained single-objective optimal beamforming methods. We also validate the effectiveness of our proposed method in a conference room setting. The proposed method achieves a white noise gain of 8.28 dB and a maximum sidelobe level of −23.42 dB at low frequency, while at high frequency it yields comparable directivity index results to both DolphChebyshev and SOCP methods, but outperforms them in terms of white noise gain and maximum sidelobe level, measuring 16.14 dB and −25.18 dB, respectively.

## 1. Introduction

In recent years, the beamforming technology of a spherical microphone array has emerged as a significant research area in applications involving three-dimensional sound field reception, indoor acoustic sound field analysis, direction of arrival (DOA) estimation, and noise control. Compared to classical linear arrays, rectangular arrays, and circular arrays, the spherical array offers ease in spatial filtering or beamforming. It can be effectively designed to enhance target sources in arbitrary directions and leverages the elegant mathematical framework of spherical harmonic transformation for array processing [1]. In practical scenarios, oversampling is often employed by spherical microphone arrays to obtain samples. By utilizing the spherical Fourier transform technique, these samples can be transferred to the spherical harmonic domain for more computationally efficient processing compared to the spatial domain. Additionally, taking advantage of decoupling between frequency components and angle components in the spherical harmonic domain allows the convenient design of wideband beamformers [2].

In this digital age, the importance of acoustic signal processing is becoming increasingly prominent. Microphone array signal processing is an indispensable technology in acoustic signal processing, which can be used in various fields such as speech recognition, human–computer interaction, and smart speakers. Therefore, this article aims to explore the application of advanced technologies such as microphone array beamforming to meet the growing communication and perception needs of modern times. It is worth mentioning that there are some interesting research results worth noting recently, such as Dong et al. [3] proposed an efficient source localization method and applied it to mining engineering, S. Cantero-Chinchilla et al. [4] applied beamforming technology to damage localization, and Allegro, G [5] designed and implemented a novel acoustic system that uses only low-cost off-the-shelf hardware and transmits a single appropriately designed signal in an inaudible frequency range to perform integrated perception and communication.

When random interference signals impinge on the receiving array, the signal processing system typically employs the adaptive null steering algorithm in the preprocessing stage to mitigate these interferences. However, the convergence speed and effectiveness of this algorithm often fall short, resulting in significant performance degradation. Therefore, designing a beamformer capable of effectively suppressing dynamic interference from sidelobe regions remains an active research area.

With the spherical harmonic expansion and orthogonality of spherical functions, we can calculate the array output in the spherical harmonic domain. Performing calculations in this domain has distinct advantages as we only need to adjust the array modal strength to model directional vectors for various array configurations. Rafaely [6] utilized the delay caused by a single plane wave as beamforming weights and successfully designed a delay-and-sum beamformer in the spherical harmonic domain. This beamformer exhibits high robustness, but performs poorly in terms of directionality at low frequencies. The conventional beamformer proposed by Li and Duraswami [7], which features constant array weights, has been widely applied in the field of plane wave decomposition [8]. Rafaely introduced a beamformer with maximum white noise gain in [9], which is equivalent to a delay-and-sum beamformer in free-field environments, which further proves the reason why delay-and-sum beamformers are widely used, as they possess reliable and robust characteristics. YuKang Liu proposed a superdirective beamformer in [10] that achieves maximum directional gain. However, achieving a high directional index may come at the expense of robustness. The aforementioned methods do not strive to strike a balance between these two aspects. For this purpose, various design methods for beamformers with mixed objectives have been proposed. For instance, Rafaely [9] presents a design method for beamformers with mixed objectives, which achieves a natural balance between directionality and white noise gain [11]. Meyer and Elko [12], presented array weight optimization methods to find the balance between beamforming directivity and robustness, which is useful in practical applications. However, these methods lack the capability to exert control over the sidelobe level of the beam pattern. Rafaely et al. also employed the classic Dolph–Chebyshev beam mode design method (DolphChebyshev) [13] in the spherical harmonic domain to address this issue; however, this approach neglects consideration of white noise gain control, resulting in reduced robustness of low-frequency designed beamformers. Although Shefeng Yan and U. Peter Svensson et al. simultaneously considered multiple conflicting performance indicators, the weight vector design problem of the beamformer in the spherical harmonic domain was formulated as a multi-constraint problem to control various performance indicators such as sidelobe level (SOCP) [14,15]. However, this approach primarily optimizes a single target, limiting its ability to achieve overall optimality. Additionally, determining appropriate constraint values poses challenges for this method and requires advanced theoretical knowledge and engineering experience from users. Table 1 summarizes the advantages and disadvantages of the two aforementioned beamforming design methods capable of controlling sidelobe levels. While the studies mentioned above considered only symmetrical beampatterns, Rafaely [16] extended the beampattern design methods to non-symmetric cases for a spherical microphone array. The approach has been devised for both the spatial and spherical harmonics domains, utilizing a multiple null-steering method. This method creates notches in the beampattern and directs them towards interferences originating from known external beam directions, with the aim of improving the signal-to-noise ratio. Metaheuristic algorithms are widely used to solve the problem of high sidelobe levels in collaborative beamforming, as derivative-based optimization techniques often become stuck in local optima, and exhaustive search algorithms can be time-consuming [17]. In references [18,19], particle swarm optimization (PSO) algorithm and genetic algorithm (GA) are, respectively, applied to solve the problem of beamforming pattern optimization. Suhanya Jayaprakasam proposed a beam mode optimization method based on multi-objective NSGA [20]. This method effectively balances trade-offs between conflicting indicators and facilitates optimal beamformer design. Moreover, it eliminates the need for manual setting of constraint parameters in traditional methods, making it more user-friendly to implement. Overall, this approach significantly improves sidelobe suppression and directivity.

To date, the multi-objective optimization method for beam mode in the spherical harmonic domain has received limited attention. Therefore, this study fully exploits the advantages of beam design in the spherical harmonic domain and proposes a wideband beamformer design approach based on spherical harmonic domain-assisted NSGA-II [21], building upon existing literature research. The proposed method formulates the optimization problem of beam mode in the spherical harmonic domain as a constrained multi-objective optimization problem and employs the NSGA-II algorithm with constraint processing technology [22] to solve it. We also achieved dynamic control of the optimization range of beam weights by utilizing the positive-definite property of the expressions for white noise gain and directivity index. Our approach for beamformer design in the spherical harmonic domain is different from traditional methods in that it simultaneously optimizes three performance indicators: white noise gain, directional index, and maximum sidelobe level. As a result, this method provides superior overall performance for the designed beamformers. Furthermore, our proposed method requires only the pre-setting of the lowest thresholds for the white noise gain and directional index, respectively, to determine the range of optimized beamforming weights. Consequently, a series of optimal sets of beamforming weights can be obtained. So, dynamic weight selection is offered based on diverse application requirements.

The remaining sections of this paper are structured as follows: Section 2 provides an introduction to the background of the spherical Fourier transform and beamformers in the spherical harmonic domain. Section 3 presents a discussion on formulating the optimization problem for beam pattern in the spherical harmonic domain as a constrained multi-objective optimization problem, along with an algorithm employed for its solution. In Section 4 and Section 5, simulations and real-world experiments are conducted to validate the proposed method’s performance, respectively. Finally, Section 6 concludes this paper.

## 2. Background

The present study adopts the conventional Cartesian coordinate system (x,y,z) and the spherical coordinate system (r,θ,ϕ), where the elevation angle θ and azimuth angle ϕ are measured in radians from the positive *z*-axis and positive *x*-axis, respectively. Considering a unit amplitude plane wave arriving from direction $\Omega_{o}=(\theta_{o},\phi_{o})$ with a wavenumber k, impinging on a spherical array with a radius a and M microphones mounted on its surface; the sound field of the plane wave at a point $\Omega_{o}=(\theta_{o},\phi_{o})$ on the surface of the sphere can be expressed as follows [23,24]:

(1) $$p(ka,\Omega_{o},\Omega_{k})=\sum_{n=0}^{\infty }\sum_{m=-n}^{n}b_{n}(ka){\left[Y_{n}^{m}(\Omega_{k})\right]}^{*}Y_{n}^{m}(\Omega_{o}),$$

where $Y_{n}^{m}$ represents the spherical harmonic function of order n and degree m; * denotes the complex conjugate, $k=\frac{2\pi f}{c}$ denotes the wavenumber relative to the speed of sound c, and $b_{n}(ka)$ signifies the mode strength of the spherical array, which is contingent upon the array configuration. The commonly employed array configurations include open and rigid spherical arrays, with their corresponding mode strengths determined by equation [24].

(2) $$b_{n}(ka)=\left\{\begin{array}{ll} 4\pi i^{n}j_{n}\left(ka\right) & open\;sphere\\ 4\pi i^{n}\left[j_{n}\left(ka\right)-\frac{j_{n}^{\prime }\left(ka\right)}{h_{n}^{\prime }\left(ka\right)}h_{n}\left(ka\right)\right] & grid\;sphere \end{array}\right.,$$

where $i=\sqrt{-1}$ is an imaginary unit; $j_{n}$ and $h_{n}$ are the nth-order spherical Bessel and Hankel functions, respectively; $j_{n}^{\prime }$ and $h_{n}^{\prime }$ are their derivatives with respect to their arguments, respectively. The spherical harmonics, which serve as solutions to the Helmholtz equation, are defined as follows [25]:

(3) $$Y_{n}^{m}(\theta ,\varphi )=\sqrt{\frac{(2n+1)(n-m)!}{4\pi (n+m)!}}P_{n}^{m}(\cos(\theta ))e^{im\phi },$$

where $P_{n}^{m}(.)$ represents the associated Legendre functions. The spherical harmonics are a set of standard orthogonal functions that satisfy the following properties:

(4) $$\int_{0}^{2\pi }\int_{0}^{\pi }Y_{n^{\prime }}^{m^{\prime }}(\Omega )[Y_{n}^{m}(\Omega ){]}^{*}\sin\theta d\theta d\phi =\delta_{nn^{\prime }}\delta_{mm^{\prime }},$$

where $\delta_{nn^{\prime }}$ and $\delta_{mm^{\prime }}$ are Kronecker delta functions.

The spherical Fourier transform of a square integrable function p on the unit sphere, denoted as $p_{nm}$, and its corresponding inverse transform can be expressed as [26]

(5) $$p_{nm}(ka,\Omega_{k})=\int_{0}^{2\pi }\int_{0}^{\pi }p(ka,\Omega_{k},\Omega )[Y_{n}^{m}(\Omega ){]}^{*}\sin\theta d\theta d\phi ,$$

(6) $$p(ka,\Omega_{k},\Omega )=\sum_{n=0}^{\infty }\sum_{m=-n}^{n}p_{nm}(ka,\Omega_{k})Y_{n}^{m}(\Omega ).$$

The application of the spherical Fourier transform (5) to a plane wave, as represented by Equation (1), yields the expression in the spherical harmonic domain for $p(ka,\Omega_{o},\Omega_{k})$ as follows:

(7) $$p_{nm}(ka,\Omega_{k})=b_{n}(ka)[Y_{n}^{m}(\Omega_{k}){]}^{*}.$$

Note that for simplicity, $p_{nm}(ka,\Omega_{k})$ is sometimes also written as $p_{nm}(ka)$.

If we denote the aperture weighting function by w, the array output is given as the integral of the product between the array input signal and the complex conjugated weighting function over the entire sphere [2]:

(8) $$\begin{array}{ll} y\left(ka\right)= & \int_{0}^{2\pi }\int_{0}^{\pi }p\left(ka,\Omega \right)w^{*}\left(k,\Omega \right)\sin\theta d\theta d\phi \\ & =\sum_{n=0}^{\infty }\sum_{m=-n}^{n}p_{nm}(ka)w_{nm}^{*}(k) \end{array},$$

where $w_{nm}$ denotes the spherical Fourier transform coefficients of w.

In practice, the sound pressure is spatially sampled at microphone positions $\Omega_{s}$, where s=1,...,M. The positioning of the microphones must adhere to the following discrete orthogonality condition:

(9) $$\sum_{s=1}^{M}a_{s}Y_{n^{\prime }}^{m^{\prime }}(\theta_{s},\phi_{s})[Y_{n}^{m}(\theta_{s},\phi_{s}){]}^{*}=\delta_{nn^{\prime }}\delta_{mm^{\prime }},$$

where $a_{s}$ is a real number determined by the sampling scheme, and for near uniform sampling, we have $a_{s}=\frac{4\pi }{M}$.

To avoid spatial aliasing and achieve accurate sound field reconstruction, the number of microphones must satisfy $M\geq (N+1{)}^{2}$, and the reconstruction order must satisfy N≥ka. A further analysis of the aliasing error in spherical sampling can be found in [27].

The discrete spherical Fourier transform of $p(ka,\Omega_{k})$ and the inverse transform are given by

(10) $$p_{nm}(ka,\Omega_{k})=\sum_{s=1}^{M}a_{s}p(ka,\Omega_{s},\Omega_{k})[Y_{n}^{m}(\Omega_{s}){]}^{*},$$

(11) $$p(ka,\Omega_{k},\Omega )=\sum_{n=0}^{N}\sum_{m=-n}^{n}p_{nm}(ka,\Omega_{k})Y_{n}^{m}(\Omega ).$$

Rafaely introduced multiple spatial sampling schemes in [2]. For the sake of simplification, we assume in this paper that the microphones are uniformly distributed on the surface of the sphere.

The corresponding array output becomes

(12) $$y(ka)=\sum_{s=1}^{M}a_{s}p(ka,\Omega_{s})w^{*}(k,\Omega_{s})\;=\sum_{n=0}^{N}\sum_{m=-n}^{n}p_{nm}(ka)w_{nm}^{*}(k).$$

where $w^{*}(k,\Omega_{s})$ denotes the array weights and $w_{nm}^{*}(k)$ denotes their spherical Fourier coefficients.

Meyer and Elko proposed a beamforming weight expression in the spherical harmonic domain, which yields beampatterns that are axisymmetric when viewed from an axis of symmetry [27] and is given by the following expression:

(13) $$w_{nm}^{*}(k)=\frac{d_{n}}{b_{n}(ka)}Y_{n}^{m}(\theta_{l},\phi_{l}),$$

where $d_{n}$ is a new real-valued beamforming weight and $\Omega_{l}=(\theta_{l},\phi_{l})$ represents the viewing direction of the array. Substituting Equations (7) and (13) into Equation (12) yields the simplified array output as

(14) $$y(\Theta )=\sum_{n=0}^{N}\sum_{m=-n}^{n}d_{n}\left(k\right)Y_{n}^{m}(\Omega_{l})[Y_{n}^{m}(\Omega_{k}){]}^{*}\;=\sum_{n=0}^{N}d_{n}\frac{2n+1}{4\pi }P_{n}(\cos(\Theta )),$$

where Θ is the angle between $\Omega_{l}$ and $\Omega_{k}$. The derivation of the above equation uses the addition theorem of spherical harmonics [28], which is shown below:

(15) $$\sum_{m=-n}^{n}Y_{n}^{m}(\Omega_{l})[Y_{n}^{m}(\Omega_{k})]*=\frac{2n+1}{4\pi }P_{n}(\cos(\Theta )).$$

Equation (14) can be written in the following matrix form:

(16) $$y(\Theta )=d_{n}^{T}v_{n}(\Theta ),$$

where

(17) $$d_{n}=[d_{0},d_{1},...,d_{N}{]}^{T},$$

(18) $$v_{n}(\Theta )=\frac{1}{4\pi }[P_{0}(\cos(\Theta )),3P_{1}(\cos(\Theta )),...,(2N+1)P_{N}(\cos(\Theta )){]}^{T}.$$

The weights $d_{n}$ now govern the response of the array’s beam pattern to unit-amplitude plane waves, and the array output y(Θ) solely relies on the incident direction of the plane wave relative to the array’s pointing direction. Consequently, it exhibits axial symmetry around the array’s pointing direction and can be conveniently rotated to other directions. Furthermore, by incorporating the $\frac{1}{b_{n}(ka)}$ term in Equation (13), it eliminates frequency-dependent components of the spherical harmonic-domain wave field in Equation (14). Thus, a set of array weights enables achieving a frequency-independent beam pattern, significantly simplifying broadband beamformer design process.

## 3. Method

The proposed metaheuristic multi-objective beamforming optimization method based on NSGA-II is presented in this section. Firstly, the formulation of the beamforming optimization problem as a multi-objective optimization problem is demonstrated. Secondly, the design concept and specific implementation details of the metaheuristic algorithm are provided.

### 3.1. Multi-Objective Beamforming Design Model

The optimization objective of this paper is to select $d_{n}$ in order to generate an optimal beamforming with a low sidelobe level, a high directional index, and a high white noise gain while maintaining an undistorted response to the array viewing direction. Subsequently, we elucidate how to formulate this objective as a constrained multi-objective optimization problem.

First, three crucial measures pertaining to array performance are presented in conjunction with the simplified Expression (16) for array output. The first measure is the white noise gain, which quantifies the improvement in the signal-to-noise ratio (SNR) at the array output compared to that at the input; a higher white noise gain indicates greater robustness of the beamformer. The formula for calculating the white noise gain is provided by [9]

(19) $$WNG=\frac{d_{n}^{T}Ad_{n}}{d_{n}^{T}Bd_{n}},$$

where

(20) $$A=v_{n}v_{n}^{H},$$

(21) $$B=\frac{4\pi }{M}diag(v_{n})\times diag(|b_{0}{|}^{-2},|b_{1}{|}^{-2},...,|b_{N}{|}^{-2}).$$

Additionally,

(22) $$v_{n}=v_{n}(0)=[1,3,..,(2N+1){]}^{T},$$

diag(.) refers to the diagonalization operation.

The second measure is the directivity index, which is defined as the ratio between the peak and average values of the squared beam pattern; a larger directivity factor indicates an enhanced directional response for the array. This expression is also given by [9]

(23) $$DF=\frac{d_{n}^{T}Ad_{n}}{d_{n}^{T}Cd_{n}},$$

where

(24) $$C=\frac{1}{4\pi }diag(v_{n}).$$

The third measure is the sidelobe level. In traditional beam optimization using convex optimization [14,15], the sidelobe region is discretized based on continuity principles. Subsequently, a constraint is imposed on the amplitude of the sidelobe level at each discretized point to control the performance of the beamformer in terms of sidelobe levels. In this study, we adopt the maximum value of sidelobe level (MSL) within the sidelobe region as our third measure, which can be mathematically formulated as follows:

(25) $$\left\{\begin{array}{c} MSL=max(20\log_{10}(y(\theta_{i},\phi_{i})))\\ \left(\theta_{i},\phi_{i}\right)\in \Omega_{SL},\;i=1,...,I \end{array}\right.,$$

where $\Theta_{SL}$ denotes the sidelobe region, and I represents the total number of discrete points within this region after discretization.

Then, combined with Equation (16), the distortionless response constraint can be formulated as follows:

(26) $$y(0)=d_{n}^{T}Ad_{n}=1.$$

The beamforming weight range determination process is finally presented. Initially, minimum thresholds for the directional factor ($\varepsilon_{a}$) and the white noise gain ($\varepsilon_{b}$) are established. These thresholds are then combined with Equation (26), resulting in the following expressions:

(27) $$\frac{1}{d_{n}^{T}Bd_{n}}\geq \varepsilon_{b},$$

(28) $$\frac{1}{d_{n}^{T}Cd_{n}}\geq \varepsilon_{a}.$$

Simplifying the above equations, we have

(29) $$B(1,1)d_{0}^{2}+B(2,2)d_{1}^{2}+...+B(N+1,N+1)d_{N}^{2}\leq \frac{1}{\varepsilon_{b}},$$

and

(30) $$A(1,1)d_{0}^{2}+A(2,2)d_{1}^{2}+...+A(N+1,N+1)d_{N}^{2}\leq \frac{1}{\varepsilon_{a}}.$$

The range of the beamforming weights, subject to constraints on white noise gain and directivity index, is ultimately determined as follows:

(31) $$h=[(\min([1/(\varepsilon_{b}*B(1,1)),1/(\varepsilon_{a}*A(1,1))]){)}^{1/2},(\min([1/(\varepsilon_{b}*B(2,2)),\;\;1/(\varepsilon_{a}*A(2,2))]){)}^{1/2},...,(\min([1/(\varepsilon_{b}*B(N+1,N+1)),\;\;1/(\varepsilon_{a}*A(N+1,N+1))]){)}^{1/2}],$$

(32) l=−h.

In the above equation, the vectors l and h represent the lower and upper bounds of the beamforming weights, respectively, min⁡(.) denotes the minimum value operation, and by adjusting the values of $\varepsilon_{a}$ and $\varepsilon_{b}$, the range of the beamforming weights $d_{n}$ can be controlled.

The beamforming optimization problem in the spherical harmonic domain can now be formulated as a multi-objective optimization problem, as presented below:

(33) $$min\;10\;\log_{10}(d_{n}^{T}Bd_{n}),10\;\log_{10}(d_{n}^{T}Cd_{n}),MSL\;s.t.y(0)=1,\;l\leq d_{n}\leq h.$$

Although the constrained multi-objective optimization problem described above does not possess a closed-form solution, it can be effectively addressed through the utilization of intelligent optimization algorithms. In this study, we employ the NSGA-II algorithm with constraint handling to tackle this problem. For ease of exposition, we denote $f_{1}$ as $10\;\log_{10}(d_{n}^{T}Bd_{n})$, $f_{2}$ as $10\;\log_{10}(d_{n}^{T}Cd_{n})$, and $f_{3}$ as MSL in subsequent discussions. Meanwhile, we define the deviation function $v=d_{n}^{T}v_{n}(0)-1$. Please note the difference between this v and the bolded v mentioned earlier, as they represent different meanings.

### 3.2. Ideas and Implementation Details of the Metaheuristic Algorithm

The Nondominated Sorting Genetic Algorithm II (NSGA-II) is a multi-objective optimization algorithm that improves and optimizes the NSGA [29]. It comprises two main components: nondominated sorting and crowding distance calculation. Nondominated sorting is a ranking method used to distinguish different levels of the Pareto front for individuals in the population, while crowding distance calculation is a technique used to ensure that the Pareto front is evenly distributed. NSGA-II has several advantages, including its ability to simultaneously handle multiple objective functions, finding a set of optimal solutions in the Pareto front, and its fast convergence speed and high efficiency.

However, it should be noted that the original NSGA-II algorithm is only suitable for ordinary multi-objective optimization problems where objective functions typically have no constraints and can be directly calculated to determine their respective Pareto fronts. As stated in Section 3.1, the multi-objective beamforming optimization problem we proposed has an equality constraint. Therefore, we introduce an adaptive penalty function and the distance measurement constraint handling technique proposed by Yonas Gebre Woldesenbet et al. [22] to address the constraint handling problem in multi-objective evolutionary algorithms. In this technique, the penalty function and distance measurement are dynamically adjusted based on the individual objective function values and constraint violation degrees. By modifying the objective function, this technique can find the optimal feasible and infeasible solutions during nondominated sorting. This approach is simple, easy to use, does not require any parameter tuning, and has shown good performance in experiments.

The specific steps of the algorithm proposed in this paper are shown as follows.

1. Determine the sidelobe range $\Omega_{SL}$, discrete sampling method and sampling points I; the configuration of the spherical array; the wavenumber k; the beamforming order N; the white noise gain threshold $\varepsilon_{b}$; the directional factor threshold $\varepsilon_{a}$; and the NSGA-II related parameters. By setting large $\varepsilon_{a}$ and $\varepsilon_{b}$ values, the range of the optimization variable $d_{n}$ can be narrowed to make the algorithm converge faster to the optimal solution.
2. Calculate the lower bound l and upper bound h of the beamforming weights to be optimized using Equations (31) and (32).
3. Initialize the population and generate the initial set of individuals.
  (34) $$d_{n}^{j}=rand(l,h),$$
4. where j=1,2,…,J, J represents the population size and $d_{n}^{j}$ represents the jth individual in the population.
5. Calculate the objective function value $f_{l}^{j}$ and constraint deviation value $v^{j}$ for each individual in the population based on Equation (33), where l=1,2,3.
6. Apply constraint handling techniques [22] to each individual to calculate the distance measure $d_{l}^{j}$ and penalty function $p_{l}^{j}$ in each objective function dimension. The specific calculation process is described in detail in reference [22]. The modified objective function value in the ith objective function dimension is the sum of the penalty function and distance measure
  (35) $$F_{l}^{j}=d_{l}^{j}+p_{l}^{j}.$$
7. Perform nondominated sorting [21] on the current population based on the modified objective function value.
8. Assign fitness to individuals based on their Pareto ranking and crowding distance.
9. Use tournament selection to select J parents.
10. Generate J offspring solutions through simulated binary crossover and polynomial mutation operations [30].
11. Combine the parent and offspring populations into a set of 2J individuals, perform nondominated sorting on this set, and select J individuals based on their fitness to form the new generation population.
12. Continue executing steps 4–10 until the maximum number of generations has been reached.

**Figure 1:**
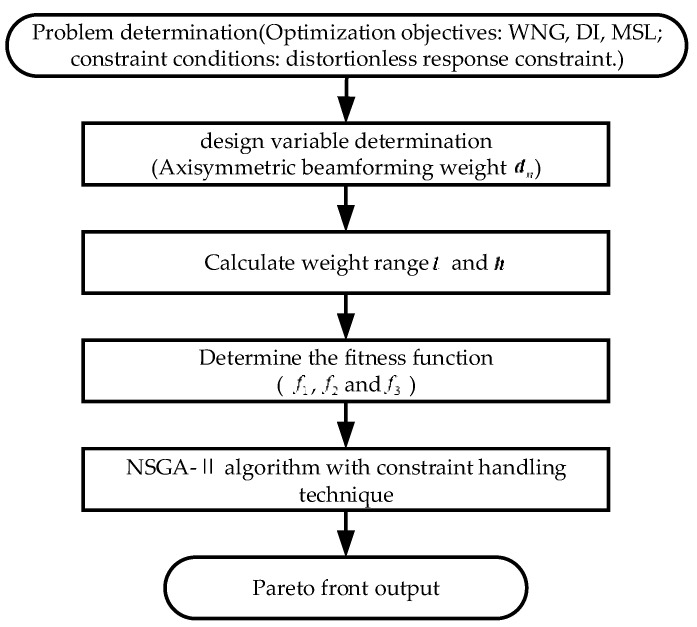
The workflow of the proposed method.

The relevant parameters of the NSGA-II algorithm in Step 1 mainly include population size, number of generations, crossover probability, mutation probability, and selection method. For population size, it is recommended to choose between 100–300 individuals. A smaller population size may result in premature convergence, while a larger population size may increase computational time. For the number of generations, it is suggested to choose a value between 1000–3000 based on the complexity of the problem and available computational resources, and adjust it based on the convergence behavior. For low-frequency problems, larger values for these two parameters can be set, while smaller values are recommended for high-frequency problems to reduce computational time. A crossover probability of 0.8 and a mutation probability of 0.1 are recommended, as higher values promote exploration and lower values promote exploitation. The optimal balance can be found through experimentation. For selection method, tournament selection is recommended, as it is a commonly used method in NSGA-II. Figure 1 shows the workflow diagram of the proposed algorithm.

## 4. Results and Discussion

This study uses both the NSGA-II algorithm with constraint handling and the multi-objective particle swarm optimization [31] (MOPSO) algorithm with constraint handling to address the beam pattern optimization problem in the spherical harmonic domain. A comparative analysis is conducted between the obtained solution and those achieved by conventional optimization algorithms capable of controlling sidelobes, namely, the Dolph–Chebyshev beampattern design method (DolphChebyshev) [13] and the optimal minimum sidelobe beamforming method in the spherical harmonic domain (SOCP) [15]. When using the SOCP method, our goal is to maximize the directionality of the array while satisfying the preset white noise gain constraint, distortion-free response constraint and sidelobe level constraint. We determine the values of these constraint parameters using the optimization results of the proposed algorithm as a guiding principle.

The output of the algorithm proposed in this paper is a set of feasible optimal solutions. Therefore, for subsequent simulations and measurements, we select the solution from this set that has the minimum Euclidean distance to the utopia point O. The utopia point is defined as follows:

(36) $$O=\left[f_{1min},f_{2min},f_{3min}\right]=[\underset{a\in Q}{min}\left(f_{1a}\right),\underset{b\in Q}{min}\left(f_{1b}\right),\underset{c\in Q}{min}\left(f_{1c}\right)],$$

where $f_{1a}$ represents the value of solution a with respect to the objective function $f_{1}$, and so on; Q represents the set of solutions output by the proposed algorithm. The Euclidean distance between solution q and the utopian point O is defined as follows:

(37) $$d_{q}=\sqrt{{\left(f_{1q}-f_{1min}\right)}^{2}+{\left(f_{2q}-f_{2min}\right)}^{2}+{\left(f_{3q}-f_{3min}\right)}^{2}}.$$

All simulations and results were derived with the following parameter settings: Rigid spheres equipped with 32 and 36 microphones, uniformly distributed across their surfaces, are employed for third-order and fourth-order beamforming in the spherical harmonic domain, respectively. For NSGA-II, the crossover index is set to $\eta_{c}=20$, the mutation index is set to $\eta_{m}=100$, the mutation probability is set to 0.2, the number of generations is set to 3000, and the population size is set to 400. For the MOPSO algorithm, both the population and repository size are set to 200. The inertia weight, personal learning coefficient, global learning coefficient, and mutation rate are set to 0.5, 1, 2, and 0.4, respectively. The array’s viewing direction is $\Omega_{l}=(0^{\circ },90^{\circ })$, and the sidelobe region is uniformly sampled at intervals of $2.4^{\circ }$. The WNG and DF thresholds for the proposed algorithm are set to $\varepsilon_{a}=\varepsilon_{b}=1$. 

### Sample Results of the Optimization Process

**Figure 2:**
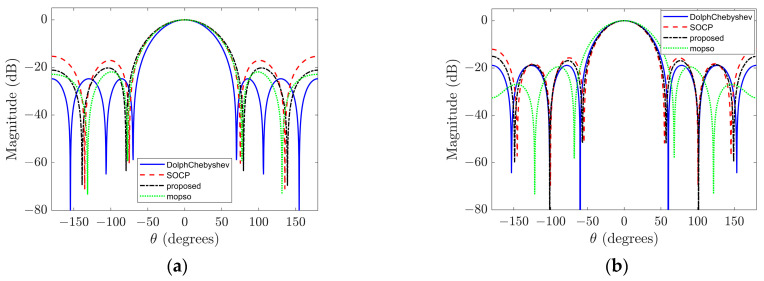
Sampled beampatterns at different frequencies. (**a**) Sampled beampattern at ka=1; (**b**) sampled beampattern at ka=2.

**Figure 3:**
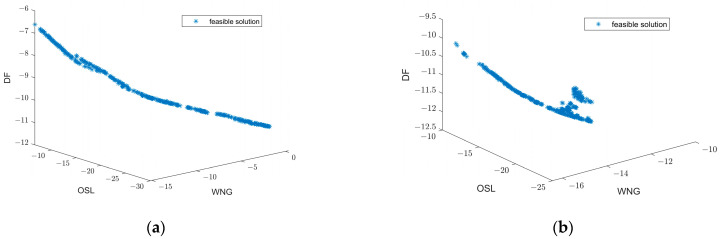
Pareto optimal front solutions for sample distribution depicted in Figure 2. (**a**) ka=1; (**b**) ka=2.

Firstly, considering a third-order rigid sphere array, Figure 2a,b illustrate the beampatterns obtained using different beamforming design methods at low frequency (ka=1) and high frequency (ka=2), respectively. At a low frequency, for the Dolph–Chebyshev method, the main-lobe width is set to 70°; for the SOCP method, the minimum WNG constraint is set to 5 and the maximum sidelobe level constraint is set to −10 dB; additionally, the sidelobe region $\Omega_{sl}=\{(\theta ,\phi )|70^{\circ }\leq \left|\phi \right|\leq 180^{\circ },\theta =90^{\circ }\}$ is defined. Similarly, at a high frequency using the Dolph–Chebyshev method with a main-lobe width of 60°; in SOCP method with minimum WNG constraint of 10 and maximum sidelobe level constraint of −10 dB; also defining sidelobe region as $\Omega_{sl}=\{(\theta ,\phi )|60^{\circ }\leq \left|\phi \right|\leq 180^{\circ },\theta =90^{\circ }\}$. Table 2 provides a comparison of these beampatterns’ main features where optimal DI, WNG, and MSL values are highlighted in bold for each sample within both frequencies. The Pareto optimal front solutions for the sample distribution depicted in Figure 2 are illustrated in Figure 3. The figure reveals that these Pareto optimal solutions are predominantly distributed along a curve, thereby indirectly validating the effectiveness of the proposed methodology and furnishing a dependable basis for dynamically selecting beamforming weights based on application requirements.

The results depicted in Figure 2 and Table 2 demonstrate that at higher frequencies, it is possible to achieve a beamformer with a narrower main lobe width while simultaneously maintaining or even enhancing other performance indicators compared to lower frequencies. At high frequency, the proposed method achieves a DI value decrease of only 0.0391 dB compared to the SOCP method, while increasing the WNG and MSL values by 0.676 dB and 2.983 dB, respectively. Similarly, at low frequency, the proposed method achieves only slight decreases in DI and WNG values (0.029 dB and 0.0193 dB, respectively), but increases the MSL value by 1.8437 dB compared to the SOCP method. Overall, the proposed method significantly improves the maximum sidelobe level compared to the SOCP method while almost maintaining other performance indicators at both high and low frequencies. Additionally, the proposed method does not require a complex constraint parameter tuning process compared to the SOCP method. Although the computational complexity of the proposed algorithm is high, these complex calculations can be completed offline, so there is no impact on the practical application of the algorithm. When using NSGA-II and MOPSO algorithms as optimization algorithms for beamforming, the former performs better in terms of white noise gain at low frequency, while the latter performs better in terms of maximum sidelobe level. Meanwhile, at high frequency, the MOPSO algorithm achieves improvements in WNG and MSL values at the expense of a wider main lobe width and a smaller DI value. Therefore, we cannot determine which optimization algorithm is better overall. However, later on, we will see that when the beamforming order is four, the NSGA-II algorithm outperforms the MOPSO algorithm as the optimization algorithm.

**Figure 4:**
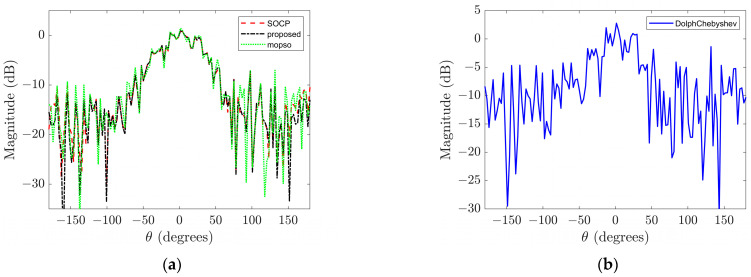
The beampatterns under the existence of 15 dB noise in the input signal at low frequency. (**a**) SOCP and proposed methods; (**b**) Dolph–Chebyshev method.

However, it should be noted that the performance enhancement achieved by the Dolph–Chebyshev method comes at the expense of a wider main lobe width; specifically, the former beampatterns exhibit a main lobe width of 60° whereas the latter has a narrower width of only 56°. Similarly, at low frequency, although the beampatterns obtained through the Dolph–Chebyshev method demonstrate superior DI and MSL values among all three methods considered here, this advantage is accompanied by compromised WNG performance. In particular, at low frequency, the beampatterns obtained by the Dolph–Chebyshev method have a −0.6950 dB WNG value, resulting in very poor noise robustness of the array [15]. Figure 4 shows the beampatterns corresponding to the input signal with a SNR of 15 dB at low frequency. It can be observed from the figure that the beampatterns obtained by the Dolph–Chebyshev method is severely degraded, while the beampatterns corresponding to the other two algorithms well retains the original form.

**Figure 5:**
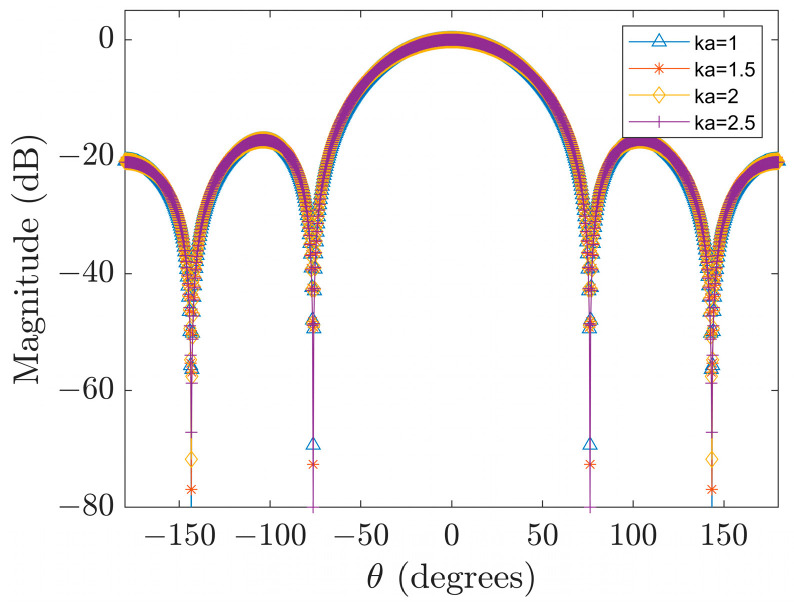
Frequency-independent beampattern.

Finally, as shown in Equation (16), the frequency-dependent component has been removed in advance, so only a set of array weights is required to achieve a frequency-independent beampattern. This is one of the main advantages of designing a broadband beamformer in the spherical harmonic domain compared with the spatial domain [32]. Figure 5 shows the frequency-independent beampatterns generated by the weights obtained at ka=1 using the proposed algorithm.

**Figure 6:**
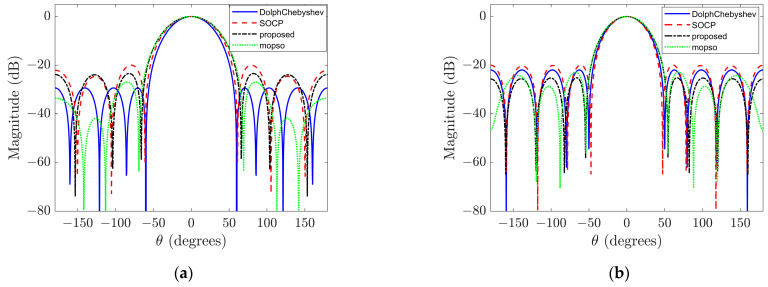
Sampled beampatterns of fourth order at different frequencies. (**a**) Sampled beampattern at ka=1.5; (**b**) sampled beampattern at ka=3.

In order to verify the effectiveness of the proposed algorithm, we choose a rigid spherical array of order N=4, and compare the proposed algorithm with the DolphChebyshev method and SOCP method at low frequency (ka=1.5) and high frequency (ka=3) again. At low frequency, for the DolphChebyshev method, we set the main lobe width to 60°, for the SOCP method, we impose a minimum white noise gain constraint of 5 dB and a maximum side lobe level constraint of −20 dB. The sidelobe region is defined as $\Omega_{sl}=\{(\theta ,\phi )|60^{\circ }\leq \left|\phi \right|\leq 180^{\circ },\theta =90^{\circ }\}$. At high frequency, for the Dolph–Chebyshev method, we set the main lobe width to 50°, for the SOCP method, we impose a minimum white noise gain constraint of 15 dB and a maximum sidelobe level constraint of −20 dB. The sidelobe region is defined as $\Omega_{sl}=\{(\theta ,\phi )|50^{\circ }\leq \left|\phi \right|\leq 180^{\circ },\theta =90^{\circ }\}$. The corresponding beampatterns are presented in Figure 6. The major characteristics of these beampatterns are compared in Table 3. From Table 3(b), it can be seen that the proposed algorithm only has a slight loss in DI value compared to the two traditional algorithms under high frequency, but there is a significant improvement in WNG and MSL. When using NSGA-II as the optimization algorithm to solve the optimization problem, it outperforms MOPSO on both DI and MSL, with only a slight loss on WNG. It can be seen from Table 3a that, at a low frequency, the beamforming is similar to that of the third-order rigid spherical array. Although the Dolph–Chebyshev method achieves optimal DI and MSL values, it comes at the expense of sacrificing array’s noise robustness. Meanwhile, under low frequency, compared with the SOCP method and the MOPSO method, the proposed algorithm only has a slight loss in DI value, but has significant improvement in WNG and MSL. Meanwhile, using MOPSO as the optimization algorithm produces better results than two traditional algorithms.

## 5. Experimental Results

**Figure 7:**
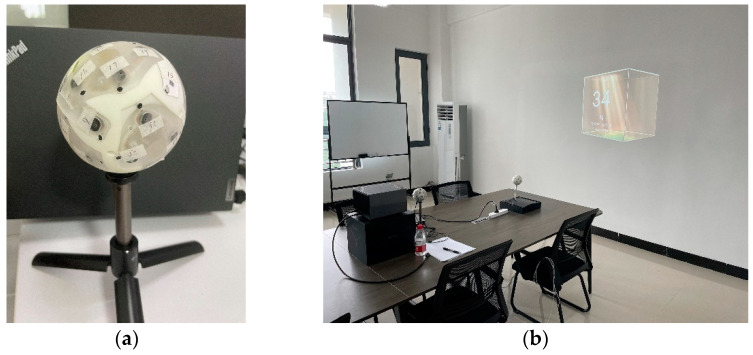
Experimental setup. (**a**) Spherical microphone array; (**b**) conference room.

**Figure 8:**
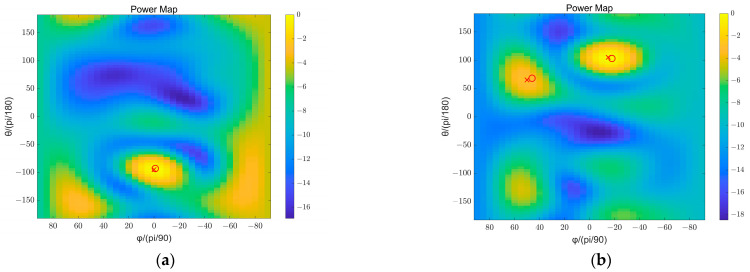
Energy spectrum at 3890.625 Hz obtained using the proposed method. (**a**) Single source; (**b**) two sources.

In this section, we used real recordings recorded in a conference room to verify the effectiveness of the proposed algorithm. The spherical microphone array and test site used in the experiment are shown in Figure 7. Initially, a rigid spherical array with 32 microphones uniformly distributed on its surface was used to separately record a single stationary active sound source located at (−93°,−1°) and two stationary sound sources occurring simultaneously located at (103°,−18°) and (68°,46°). The recording time was 4 s with a sampling rate of 48 kHz. Subsequently, the collected time-domain signal was transformed into the frequency domain using Short-Time Fourier Transform (STFT). The STFT-related parameters include: a Hanning window with 1024 samples, overlapping between adjacent frames set at 50%, and a FFT length of 1024 samples. Next, we selected the data located at the frequency bin of 3890.625 Hz and carried out third-order spherical Fourier transform on it to obtain the corresponding spherical harmonic coefficients. These coefficients were then used to calculate the covariance matrix. Finally, by applying beamforming weights acquired in Section Four for high-frequency third-order rigid spherical arrays configuration, we weighted the covariance matrix and generated energy spectrum at 3890.625 Hz as illustrated in Figure 8 (the resolution of the beam scanning is 5 degrees). According to Figure 8, we can see that there is a peak in the spectrogram near the direction of each real sound source, indicating that we have successfully localized the speaker’s position. At the same time, in addition to the speaker location, there are also some higher energy regions in other directions, which were caused by early reflection of the sound source. Table 4 shows the error between the estimated and actual values of the direction.

## 6. Conclusions

A multi-objective NSGA-II wideband beamforming method based on spherical harmonic assistance is proposed, which holds promising application prospects in acoustic research fields such as multi-source positioning and tracking, speech enhancement, and intelligent speech recognition. In this paper, we transform the optimization problem of beampatterns in the spherical harmonic domain into a constrained multi-objective optimization problem with variable optimization range. We propose a method to simultaneously optimize the white noise gain, directional index, and sidelobe level during the modal beamforming process. Compared with existing methods, our proposed approach not only maintains robustness but also achieves superior results in terms of sidelobe level and directionality without requiring a complex parameter tuning process. Our current discussion is mainly focused on the problem of axisymmetric beamforming in the spherical harmonic domain. In the future, we plan to expand our approach to non-axisymmetric beamforming in the same domain.

## 7. Patents

The findings of this study have been utilized to file an invention patent, with the application number 202310363719.0.2.

## Figures and Tables

**Table 1:** Comparison of beamforming design methods capable of performing sidelobe control.

Algorithm	Advantage	Disadvantage
SOCP	Multi-objective design, moderate computational complexity.	No closed-form solution, cannot achieve Pareto optimality, complex constraint parameter settings.
DolphChebyshev	sidelobe control, closed-form solution, low computational complexity.	Single objective design, poor robustness at low frequencies.

**Table 2:** Comparison between the characteristic beampatterns in Figure 2. (**a**) ka=1. (**b**) ka=2.

**(a)**			
**Design**	**DI (dB)**	**WNG (dB)**	**MSL (dB)**
DolphChebyshev	11.3026	−0.6950	−24.7364
SOCP	10.0305	6.9897	−15.3544
MOPSO (proposed)	10.3674	4.5828	−21.8563
Proposed	10.0093	6.6868	−20.1317
(**b**)			
**Design**	**DI (dB)**	**WNG (dB)**	**MSL (dB)**
DolphChebyshev	11.8739	12.5796	−18.8057
SOCP	12.0412	11.1646	−12.0431
MOPSO (proposed)	11.1404	15.1728	−19.4403
Proposed	12.0021	11.8406	−15.0269

**Table 3:** Comparison between the characteristic beampatterns in Figure 6. (**a**) ka=1.5; (**b**) ka=3.

**(a)**			
**Design**	**DI (dB)**	**WNG (dB)**	**MSL (dB)**
DolphChebyshev	12.9701	−0.9677	−29.3730
SOCP	12.0397	6.9898	−20.0015
MOPSO (proposed)	11.8018	6.3249	−21.7587
Proposed	11.6908	8.2891	−23.4221
(**b**)			
**Design**	**DI (dB)**	**WNG (dB)**	**MSL (dB)**
DolphChebyshev	13.7193	15.2087	−21.9742
SOCP	13.8492	14.5303	−20.5303
MOPSO (proposed)	13.3070	16.5182	−22.9061
Proposed	13.3978	16.1437	−25.1887

**Table 4:** The result of the beam scanning.

Scenario	Ground Truth DOA	Estimated DOA	Error
Single source	(−93°,−1°)	(−95°,0°)	1°
Dual source	(103°,−18°)	(105°,−15°)	3.0529°
	(68°,46°)	(65°,50°)	4.5785°

## Data Availability

Not applicable.
